# Cutaneous Squamous Cell Carcinomas in Organ Transplant Recipients

**DOI:** 10.3390/jcm4061229

**Published:** 2015-06-03

**Authors:** Ramya Chockalingam, Christopher Downing, Stephen K. Tyring

**Affiliations:** 1 Medical School, the University of Texas Health Science Center at Houston, 6431 Fannin, Houston, TX 77030, USA; 2 Center for Clinical Studies, 1401 Binz, Suite 200, Houston, TX 77004, USA; E-Mails: cdowning@ccstexas.com (C.D.); styring@ccstexas.com (S.K.T.); 3 Department of Dermatology, University of Texas Health Science Center at Houston, Houston, TX 77030, USA

**Keywords:** human papillomavirus (HPV), organ transplant, skin cancer, squamous cell carcinoma

## Abstract

Non-melanoma skin cancers represent a major cause of morbidity after organ transplantation. Squamous cell carcinomas (SCC) are the most common cutaneous malignancies seen in this population, with a 65–100 fold greater incidence in organ transplant recipients compared to the general population. In recent years, human papillomaviruses (HPV) of the beta genus have been implicated in the pathogenesis of post-transplant SCCs. The underlying mechanism of carcinogenesis has been attributed to the E6 and E7 proteins of HPV. Specific immunosuppressive medications, such as the calcineurin inhibitors and azathioprine, are associated with a higher incidence of post-transplant SCCs compared to other immunosuppressive agents. Compared to other immunosuppressives, mTOR inhibitors and mycophenolate mofetil have been associated with a decreased risk of developing post-transplant non-melanoma skin cancers. As a result, they may represent ideal immunosuppressive medications in organ transplant recipients. Treatment options for post-transplant SCCs include surgical excision, Mohs micrographic surgery, systemic retinoid therapy, adjunct topical therapy, electrodessication and curettage, and radiation therapy. This review will discuss the epidemiology, risk factors, and management options of post-transplant SCCs. In addition, the underlying mechanisms of beta-HPV mediated carcinogenesis will be discussed.

## 1. Introduction

Organ transplantation is a life-saving intervention in individuals with end-stage organ disease or dysfunction. According to the Organ Procurement and Transplantation Network, 29,533 transplants were performed between January and December 2014 in the United States [1]. However, organ transplantation is not without risks. Non-melanoma skin cancers are one of the major causes of morbidity after organ transplantation [2]. They can be attributed to the immunosuppressive medications taken after transplantation, which compromise immunity and lead to an increased susceptibility of infections and malignancies. The cutaneous malignancies that develop after organ transplantation are generally more aggressive and numerous than those seen in the general population [3]. Of all the cutaneous malignancies, squamous cell carcinoma (SCC) is the predominant type, with a 65–100 fold increased incidence in transplant recipients compared to the general population [3]. In recent years, a role for HPV in cutaneous malignancy has been proposed.

Papillomaviruses are double-stranded, circular DNA viruses that can infect cutaneous or mucosal epithelium, where they can cause neoplasms or remain asymptomatic [4]. Of the 189 types that have been classified, it is estimated that more than 120 infect humans [5]. HPV’s oncoproteins, E6 and E7, which respectively inhibit the p53 and pRb tumor suppressor proteins, play a major role in the carcinogenesis of these viruses. The beta-papillomaviruses, such as HPV5, HPV8, and HPV9, are responsible for preneoplastic and neoplastic skin lesions, particularly among immunocompromised individuals. As such, these viruses are detected in up to 90% of the cutaneous SCCs observed in transplant recipients [6].

## 2. Epidemiology and Risk Factors

Cutaneous squamous cell carcinomas (SCC) are the most common malignancy seen in organ transplant recipients. Clinically, they present as infiltrating, nodular lesions with ulceration or bleeding, and they generally occur on sun-exposed areas [7]. Before age 40, the majority of the lesions are seen on the dorsum of the hands, the forearms, and the trunk. After age 40, the majority of lesions are present in the head and neck area [7]. The risk of cutaneous SCC increases over time, with the incidence ranging from 10%–27% at 10 years post-transplant and 40%–60% at 20 years post-transplant in the United States and western Europe [8]. Other less common cutaneous malignancies seen in organ transplant patients include basal cell carcinomas (BCC), Merkel cell carcinomas, and Kaposi’s sarcoma.

Individuals with heart or lung transplants have been found to have a higher incidence of cutaneous SCC compared to individuals with kidney or liver transplants [9]. This is presumed to be a result of both the more intensive immunosuppressive regimens needed after heart and lung transplants as well as the older age of patients receiving these transplants [8]. Liver transplants have been associated with a lower incidence of cutaneous SCC compared to other organ transplants [8]. Some studies have found that liver transplant recipients actually have a greater incidence of cutaneous BCCs compared to SCCs [10].

The cutaneous SCCs in organ transplant recipients express a different molecular phenotype than those in immunocompetent adults. These variations include increased expression of p53 and transforming growth factor-beta and decreased expression of phosphorylated mTOR and p70S6K [6]. Furthermore, a spindle cell component is present in 20% of cutaneous SCCs in transplant patients, indicating epithelial-to-mesenchymal transition as a result of immunosuppression [6]. Interestingly, SCCs of the spindle cell type are exceedingly rare in the general population.

Risk factors for the development of cutaneous SCCs in organ transplant recipients include sun exposure, increased sensitivity to UV light (fair skin, blue eyes, and red hair), male sex, older age, smoking, living in subtropical regions, and a history of severe sunburns [11,12]. Furthermore, the immunocompromised nature of the transplant patient impairs immune surveillance, which is conducive to precancerous changes [13]. Greater ages at transplantation and increased intensity and duration of immunosuppressive regimens have also been identified as risk factors [14]. Specific immunosuppressive medications, such as cyclosporine and azathioprine, have been linked to the development of skin cancer. Cyclosporine, a calcineurin inhibitor, promotes tumor invasiveness and stimulates tumor growth via VEGF-mediated angiogenesis [3]. Cyclosporine also inhibits DNA repair mechanisms, thereby preventing repair of ultraviolet-induced skin damage [3]. Azathioprine, an antimetabolite, sensitizes DNA to ultraviolet-A radiation and promotes carcinogenesis [14]. Furthermore, voriconazole, an antifungal agent used to treat invasive aspergillosis in organ transplant recipients, has been associated with aggressive and multifocal non-melanoma skin cancers. In one study, 42.9% of lung transplant patients treated with voriconazole developed skin cancer in contrast to 9.9% of patients who did not receive voriconazole [8].

In recent years, HPV has been associated with skin cancers in organ transplant recipients. In post-transplant patients, 80% of skin SCCs are associated with HPV in contrast to only 40% in immunocompetent individuals. Specifically, the beta genus has been implicated in the development of post-transplant SCCs. The beta genus is pervasive in the general population, as beta-HPV colonizes the skin within a few days of life and increases in prevalence with age [15]. Certain risk factors, such as white skin in men, tobacco use, and green eye color in women are associated with seroconversion to cutaneous HPV [15]. The presence of antibodies to cutaneous HPV is associated with a greater incidence of SCC, and this risk is substantially greater in patients who have developed antibodies to multiple different beta-HPV types [15]. Beta-HPV subtypes, such as HPV5, HPV8, and HPV9, lead to preneoplastic and neoplastic skin lesions in immunocompromised patients and are thought to play a causal role in post-transplant cutaneous SCCs [6]. In fact, high beta-HPV viral load in eyebrow hairs is associated with a greater risk of SCC in organ transplant recipients [15]. Additionally, the risk of SCC in organ transplant recipients is increased when both viral DNA in eyebrow hairs and antibodies to at least one type of beta-HPV are detected [15]. Beta-HPVs have been implicated in initiating the development of cutaneous squamous cell carcinomas but do not appear necessary for tumor maintenance. This is further supported by the fact that the HPV viral load is increased in actinic keratosis lesions compared to SCCs [15]. In concordance with UV irradiation, beta-HPV is thought to play a significant role in the development of non-melanoma skin cancers [16]. While the underlying mechanisms are still being investigated, the E6 and E7 proteins are thought to play a major role in the oncogenicity of beta-HPVs.

## 3. Beta-HPV Mediated Oncogenesis

Papillomaviruses are small, double-stranded DNA viruses with approximately 8000 base pairs. The HPV genome is organized similarly among the different viruses, with regulatory functions encoded by the early genes and the structural components encoded by the late genes [17]. The HPVs can be divided into five genera based on their DNA sequences [18]. These include alpha, beta, gamma, mu, and nu [19]. The alpha genus consists of approximately 30 HPV types that infect the mucosa of the genital tract or the skin [20]. The alpha-HPV types can induce non-genital skin warts, genital warts, laryngeal papillomas, cervical cancer, anogenital cancers, and oropharyngeal cancers [19]. The mucosal alpha-HPV types can be classified as low-risk or high-risk. Low-risk types 6 and 11 are associated with benign genital warts. In contrast, high-risk HPV types 16 and 18 are associated with malignant cancers [20]. Alpha-HPVs encode three oncoproteins: E5, E6, and E7; the E6 and E7 proteins are capable of immortalizing and transforming cells, leading to HPV cancers [21]. Furthermore, continuous E6 and E7 expression is necessary to maintain the malignant phenotype in alpha-HPVs [22].

The HPV types of the beta genus were first discovered in flat warts, macular red or brown achromic lesions, and in cutaneous squamous cell carcinomas of patients with epidermodysplasia verruciformis [19]. Beta-HPVs have been identified in the skin and eyebrow hairs of over 80% of healthy donors, suggesting that these viruses are ubiquitous among members of the general population [19]. At the same time, these viruses have been implicated in the preneoplastic and neoplastic skin lesions of immunocompromised individuals.

While beta-HPVs are believed to play a role in initiating the development of squamous cell skin cancers, these viruses do not appear to be necessary for tumor maintenance. This is further supported by the fact that HPV prevalence and viral load are higher in premalignant lesions, such as actinic keratoses, compared to SCCs [15]. In contrast to the alpha-HPV genomes, the beta-HPV genomes are rarely integrated in the cancer cells [23]. The prevalence of beta-HPV cancers in sun-exposed areas suggests synergism between UV light induced DNA damage and the mechanism of beta-HPV [19,24]. In normal cells, UV irradiation induced DNA damage upregulates cellular defense processes, ultimately leading to p53 activation [25]. This leads to either cell cycle arrest in order to facilitate repair or apoptosis to eliminate damaged cells [25]. However, in beta-HPV infected cells, E6 and E7 circumvent these defense mechanisms, resulting in the continued proliferation of cells and the replication of viral DNA in response to UV irradiation [25]. In HPV type 38, E6 and E7 expression induces the transcription of the ΔNp73gene, which inhibits the p53-mediated transcription of genes involved in growth suppression and apoptosis [24]. ΔNp73 accumulation results in the deregulation of UV light activated cell-cycle checkpoints, suggesting a synergistic role between UV light and HPV in skin carcinogenesis [24]. While E6 and E7 expression in the basal layer does not alter the morphology of the epidermis, it facilitates the development of SCC by chemical carcinogens or chronic UV irradiation [25]. Thus, the oncogenic properties of the beta-HPVs can be attributed to the encoded E6 and E7 proteins. However, the expression of these oncoproteins is not required to maintain the malignant phenotype of non-melanoma skin cancers [15].

The E6 proteins of HPV types: 5, 8, 20, 22, 38, 76, 92, and 96 are believed to prevent UV-induced apoptosis by degrading Bak, a proapoptotic protein [19]. In contrast to E6 in alpha-HPVs, the E6 protein in beta-HPVs does not have the ability to degrade p53 [26]. In two of the beta-HPVs, HPV5 and HPV8, the E6 protein induces degradation of p300, a histone acetyl transferase that is involved in repairing UV damage [26]. P300 degradation results in decreased levels of ataxia telangiectasia and Rad3 related (ATR) protein, a phosphoinositide 3-kinase that is involved in the signaling pathway of UV damage [26]. This leads to reduced p53 phosphorylation, resulting in reduced G1 arrest, delayed DNA repair, and an increased likelihood of double stranded breaks [19,26]. Some of the beta-HPV E6 proteins have been shown to activate telomerase, thereby extending the life span of primary keratinocytes [27,28]. The E6 protein in beta-HPVs inhibits Notch signaling by recruiting Mastermind-like protein 1, MAML1 [17,29]. As Notch functions as a tumor suppressor in epithelial cells, this inhibits keratinocyte differentiation and leads to ongoing cellular proliferation. Through its combined actions on p300, MAML1 and Notch signaling, E6 may enable beta-HPV infected cells to accumulate mutations sufficient to initiate carcinogenesis [19].

The function of beta-HPV E7 proteins is similar to the alpha-HPV E7 proteins, as they both bind the retinoblastoma protein, pRb1 [19]. Studies have shown that the E7 protein in HPV 38 inactivates the tumor suppressor pRb and thereby deregulates the G1/S phase controls [30]. Another study has demonstrated that expression of E7 in HPV8 results in polyploidy with concomitantly decreased p21 and pRb expression [31]. The HPV8 E7 gene has also been shown to alter the normal differentiation and proliferation processes in keratinocytes, resulting in migration and invasion through the underlying dermis [32]. Specifically, the expression of the extracellular proteinases, MMP-1, MMP-8, and MT1-MMP, has been found to degrade extracellular matrix and basement membrane components (collagen VII, collagen IV, and laminin V). This promotes keratinocyte migration and invasion into the underlying dermis [32]. A more recent study has determined that via E7 expression, beta-HPVs may increase the number of stem cell-like keratinocytes in early carcinogenesis. Inhibiting the differentiation of these keratinocytes increases the risk of accumulated DNA damage and malignant stem cell generation [33].

The E2 protein of HPV has also been implicated in the oncogenicity of these viruses. E2 is highly conserved among the papillomaviruses and is involved in transcription regulation, apoptosis regulation, RNA processing, ubiquitination, and intracellular trafficking [19,34]. In HPV8, E2 has displayed transforming properties in both *in vitro* and *in vivo* models, but it is thought to play a less prominent role than E6 in carcinogenesis [25]. However, the underlying mechanisms are largely unknown and further investigation is required to better understand the role of E2 in HPV-mediated skin carcinogenesis.

## 4. Treatment and Management

Once a patient develops a non-melanoma skin cancer, the subsequent risk of developing additional cancers is high. 25% of patients will develop another cancer within 13 months, and 50% will develop another skin cancer within 3.5 years [35]. Compared to the general population, organ transplant recipients experience an increased risk of SCC metastasis, with a reported incidence of 5%–7% [13]. On average, the development of metastases was seen 1.4 years after the primary skin cancer was diagnosed [36]. These patients also experience greater fatalities if these metastases are left untreated, as the three-year disease-specific survival was reported as 56% in one study [13,36]. The significant burden of non-melanoma skin cancers in organ transplant recipients highlights the importance of skin cancer prevention and timely treatment in this population.

### 4.1. Preventative Measures

Prevention can be emphasized through skin cancer education, which includes counseling on sunscreen use, sun protection, wearing protective clothing, tanning bed avoidance, and self-examination for suspicious lesions [37]. Patients are recommended to have a full-body skin examination prior to transplant and regular follow-ups following organ transplantation. The follow-up period for total body skin examination generally depends on the patient’s health status and risk factors. For patients with no skin cancer or field disease, a total body skin examination can be performed every 12 months [37]. The recommended screening interval for patients with field disease or one non-melanoma skin cancer is 3–6 months [37]. Patients with multiple non-melanoma skin cancers or high-risk squamous cell carcinoma should receive a full body skin examination every 3 months [37]. Finally, patients with a metastatic squamous cell carcinoma should be screened at 1–3 month intervals [37]. HPV vaccination may also be a potential strategy of prevention, as vaccination would improve immunity against HPV [6].

### 4.2. Management Options for Low-Risk SCC

For organ transplant patients with biopsy proven skin cancer, surgical excision with clear margins or Mohs micrographic surgery is recommended. Since surgical excision does not prevent the future development of precancerous or cancerous lesions, topical therapy is often used in conjunction with surgical therapy. Among the topical therapies, imiquimod, 5-FU, and photodynamic therapy are noninvasive and can be applied to large treatment areas [37]. Imiquimod activates a pro-inflammatory response, and thereby has both antiviral and antitumor effects [6].

While surgical excision and Mohs micrographic surgery are the preferred treatment options, aggressive electrodessication and curettage may be used when the patient experiences multiple lesions or is unable to tolerate surgical procedures [37].

Systemic retinoid therapy can be considered if a patient develops between 5–10 SCCs per year [6]. Systemic retinoids are chemopreventative, but are less efficacious compared to surgical therapy [37]. They may be used in patients who are not appropriate surgical candidates. Retinoids have also been shown to affect HPV transcription or replication and may result in the clearance of HPV skin lesions [6]. However, retinoids have many contraindications. They should not be used in women who are pregnant or who may be pregnant, patients with severe hyperlipidemia refractory to therapy, and patients with elevated liver function tests [37]. One of the disadvantages of systemic retinoids is that medication discontinuation may result in a rebound effect in which patients experience multiple SCCs in a short amount of time [37].

### 4.3. Management Options for High-Risk SCC

Mohs micrographic surgery is the recommended treatment option for high-risk lesions, since it has resulted in the highest cure rates of high-risk SCC [38]. Furthermore, Mohs surgery is advantageous in areas where tissue conservation is desirable [38]. In patients who are not surgical candidates, radiation therapy has been used. Postoperative radiation therapy can also be used after an incomplete resection or when there is accompanying lymph node or perineural spread [37].

### 4.4. Selection of Immunosuppressive Medications

In organ transplant recipients, immunosuppressive medications are necessary in order to prevent graft rejection. However, immunosuppressive regimens also increase the risk of non-melanoma skin cancer formation. As mentioned earlier, certain medications are associated with an increased risk of developing skin cancers post-transplant. These include azathioprine, an antimetabolite, and the calcineurin inhibitors, including tacrolimus and cyclosporine.

mTOR inhibitors have been associated with a decreased risk of developing non-melanoma skin cancers in comparison to calcineurin inhibitors [39]. As a result, mTOR inhibitors, such as sirolimus, may be the preferred immunosuppressive medications for organ transplant recipients. Sirolimus’s mechanism of action includes the inhibition of the mTOR signaling pathway that is important in cell cycle progression, metabolism, and protein synthesis [39]. It has been shown that mTOR deficient cells differentiate into Foxp3+ regulatory cells instead of Th1, Th2, or Th17 cells [40]. In contrast, mTOR inhibition has resulted in immunostimulatory effects in CD8 T cell production and differentiation [41]. The selective effects of mTOR inhibitors on different T cell populations may help explain the reduced incidence of non-melanoma skin cancer in organ transplant recipients treated with these medications [39]. One study in kidney transplant patients with at least one previous skin SCC demonstrated that conversion from calcineurin inhibitors to sirolimus was associated with decreased risk of subsequent cutaneous malignancies [42]. However, further studies are needed to evaluate the relationship between mTOR inhibitors and skin cancer development.

Mycophenolate mofetil, an antimetabolite, has also been associated with a lower risk of malignancy formation. One study found that patients receiving mycophenolate mofetil had a reduced incidence of skin malignancies compared to patients taking azathioprine [43].

## 5. Future Directions

As the mechanisms underlying beta-HPV mediated skin carcinogenesis are being investigated, specific molecular targets may influence the future management of post-transplant skin cancers. Since the E6 protein inhibits Notch signaling, the development of Notch agonists may play a role in reversing the proliferation of SCCs that develop after organ transplantation [6]. Furthermore, HPV vaccination may prevent the development of post-transplant SCCs. However, the efficacy of the vaccination in organ transplant recipients has not been sufficiently evaluated at this point. Studies that can assess the safety and efficacy of HPV vaccination in immunocompromised individuals may eventually lead to a reduced incidence of HPV-related skin cancers in organ transplant recipients.

## 6. Conclusions

HPV related skin cancers are a major source of morbidity in organ transplant recipients. In this review, we discuss the epidemiology, risk factors, and management options of post-transplant SCCs. Furthermore, we discuss the underlying molecular mechanism of beta-HPV mediated carcinogenesis. While much of the underlying pathogenesis is unknown, the recent discoveries that have been made may lead to specific targeted therapies that can prevent or reverse post-transplant SCCs. Overall, our understanding of post-transplant skin cancers has progressed tremendously over recent years. With further molecular studies, our understanding of the pathogenesis will only continue to grow. At the same time, several advances in the treatment and management of these malignancies have been made over recent years. It is our hope that future targeted therapies and preventative measures will allow us to better manage post-transplant SCCs in the future.

## References

[1] Organ Procurement and Transplantation Network Transplant: Transplant year by organ. http://optn.transplant.hrsa.gov/.

[2] Adami J., Gabel H., Lindelof B., Ekstrom K., Rydh B., Glimelius B., Ekbom A., Adami H.O., Granath F. (2003). Cancer risk following organ transplantation: A nationwide cohort study in Sweden. Br. J. Cancer.

[3] Geissler E.K. (2015). Skin cancer in solid organ transplant recipients: Are mTOR inhibitors a game changer?. Transplant. Res..

[4] Sampogna F., Bavinck J.N., Pawlita M., Abeni D., Harwood C.A., Proby C.M., Feltkamp M.C., Euvard S., Naldi L., Neale R.E. (2012). Factors associated with the seroprevalence of 26 cutaneous and two genital human papillomavirus types in organ transplant patients. J. Gen. Virol..

[5] Bernard H.U., Burk R.D., Chen Z., van Doorslaer K., zur Hausen H., de Villiers E.M. (2010). Classification of Papillomaviruses (PVs) Based on 189 PV Types and Proposal of Taxonomic Amendments. Virology.

[6] Connolly K., Manders P., Earls P., Epstein R.J. (2014). Papillomavirus-associated squamous skin cancers following transplant immunosuppression: One Notch closer to control. Cancer Treat Rev..

[7] Dreno B. (2003). Skin cancers after transplantation. Nephrol. Dial. Transplant..

[8] Zwald F.O., Brown M. (2011). Skin cancer in solid organ transplant recipients: Advances in therapy and management. Part I: Epidemiology of skin cancer in solid organ transplant recipients. J. Am. Acad. Dermatol..

[9] Krynitz B., Edgren G., Lindelöf B., Baecklund E., Brattström C., Wilczek H., Smedby K.E. (2013). Risk of skin cancer and other malignancies in kidney, liver, heart and lung transplant recipients 1970 to 2008—A Swedish population-based study. Int. J. Cancer.

[10] Jiménez-Romero C., Municio A.M., Medina E.M., Colina F., Domene P.O., Sanz R.G., Diaz J.C.M., de Usera M.A., Elola A.M., Gonzalez E.M. (2006). Incidence of *de novo* nonmelanoma skin tumors after liver transplantation for alcoholic and nonalcoholic liver diseases. Transplant. Proc..

[11] Madeleine M.M., Carter J.J., Johnson L.G., Wipf G.C., Davis C., Berg D., Nelson K., Daling J.R., Schwartz S.M., Galloway D.A. (2014). Risk of squamous cell skin cancer after organ transplant associated with antibodies to cutaneous papillomaviruses, polyomaviruses, and TMC6/8 (EVER1/2) variants. Cancer Med..

[12] Genders R.E., Quint K.D., de Koning M.N., Plasmeijer E.I., Feltkamp M.C., Bavinck J.N.B., Zwald F., Brown M.D. (2015). Update on our understanding of HPV as a risk factor for cutaneous squamous cell carcinoma in organ transplant recipients. Advances in Transplant Dermatology.

[13] Lloyd A., Klintmalm G., Qin H., Menter A. (2015). Skin cancer evaluation in transplant patients: A physician opinion survey with recommendations. Clin. Transplant..

[14] Vajdic C.M., van Leeuwen M.T. (2009). Cancer incidence and risk factors after solid organ transplantation. Int. J. Cancer.

[15] Accardi R., Gheit T. (2014). Cutaneous HPV and skin cancer. Presse Med..

[16] Smola S. (2014). Human papillomaviruses and skin cancer. Adv. Exp. Med. Biol..

[17] White E.A., Kramer R.E., Tan M.J.A., Hayes S.D., Harper J.W., Howley P.M. (2012). Comprehensive analysis of host cellular interactions with human papillomavirus E6 proteins identifies new E6 binding partners and reflects viral diversity. J. Virol..

[18] Doorbar J., Egawa N., Griffin H., Kranjec C., Murakami I. (2015). Human papillomavirus molecular biology and disease association. Rev. Med. Virol..

[19] Howley P.M., Pfister H.J. (2015). Beta genus papillomaviruses and skin cancer. Virology.

[20] Ghittoni R., Accardi R., Chiocca S., Tommasino M. (2015). Role of human papillomaviruses in carcinogenesis. Ecancermedicalscience.

[21] White E.A., Walther J., Javanbakht H., Howley P.M. (2014). Genus beta human papillomavirus E6 proteins vary in their effects on the transactivation of p53 target genes. J. Virol..

[22] Auewarakul P., Gissmann L., Cid-Arregui A. (1994). Targeted expression of the E6 and E7 oncogenes of human papillomavirus type 16 in the epidermis of transgenic mice elicits generalized epidermal hyperplasia involving autocrine factors. Mol. Cell. Biol..

[23] Klingelhutz A.J., Roman A. (2012). Cellular transformation by human papillomaviruses: Lessons learned by comparing high-and low-risk viruses. Virology.

[24] Dong W., Arpin C., Accardi R., Gissmann L., Sylla B.S., Marvel J., Tommasino M. (2008). Loss of p53 or p73 in human papillomavirus type 38 E6 and E7 transgenic mice partially restores the UV-activated cell cycle checkpoints. Oncogene.

[25] Viarisio D., Mueller-Decker K., Kloz U., Aengeneyndt B., Kopp-Schneider A., Grone H.J., Gheit T., Flectenmacher C., Gissmann L., Tommasino M. (2011). E6 and E7 from beta HPV38 cooperate with ultraviolet light in the development of actinic keratosis-like lesions and squamous cell carcinoma in mice. PLoS Pathog..

[26] Wallace N.A., Robinson K., Howie H.L., Galloway D.A. (2012). HPV 5 and 8 E6 abrogate ATR activity resulting in increased persistence of UVB induced DNA damage. PLoS Pathog..

[27] Bedard K.M., Underbrink M.P., Howie H.L., Galloway D.A. (2008). The E6 oncoproteins from human betapapillomaviruses differentially activate telomerase through an E6AP-dependent mechanism and prolong the lifespan of primary keratinocytes. J. Virol..

[28] Gabet A.S., Accardi R., Bellopede A., Popp S., Boukamp P., Sylla B.S., Londoño-Vallejo J.A., Tommasino M. (2008). Impairment of the telomere/telomerase system and genomic instability are associated with keratinocyte immortalization induced by the skin human papillomavirus type 38. FASEB J..

[29] Restivo G., Nguyen B.C., Dziunycz P., Ristorcelli E., Ryan R.J., Ozuysal O.Y., di Piazza M., Radtke F., Dixon M.J., Hofbauer G.F. (2011). IRF6 is a mediator of Notch pro-differentiation and tumour suppressive function in keratinocytes. EMBO J..

[30] Caldeira S., Zehbe I., Accardi R., Malanchi I., Dong W., Giarrè M., de Villiers E.M., Filotico R., Boukamp P., Tommasino M. (2003). The E6 and E7 proteins of the cutaneous human papillomavirus type 38 display transforming properties. J. Virol..

[31] Akgül B., Ghali L., Davies D., Pfister H., Leigh I.M., Storey A. (2007). HPV8 early genes modulate differentiation and cell cycle of primary human adult keratinocytes. Exp. Dermatol..

[32] Akgül B., García-Escudero R., Ghali L., Pfister H.J., Fuchs P.G., Navsaria H., Storey A. (2005). The E7 protein of cutaneous human papillomavirus type 8 causes invasion of human keratinocytes into the dermis in organotypic cultures of skin. Cancer Res..

[33] Hufbauer M., Biddle A., Borgogna C., Gariglio M., Doorbar J., Storey A., Pfister H., Mackenzie I., Akgül B. (2013). Expression of betapapillomavirus oncogenes increases the number of keratinocytes with stem cell-like properties. J. Virol..

[34] Muller M., Jacob Y., Jones L., Weiss A., Brino L., Chantier T., Lotteau V., Favre M., Demeret C. (2012). Large scale genotype comparison of human papillomavirus E2-host interaction networks provides new insights for E2 molecular functions. PLoS Pathog..

[35] Lindelöf B., Sigurgeirsson B., Gabel H., Stern R.S. (2000). Incidence of skin cancer in 5356 patients following organ transplantation. Br. J. Dermatol..

[36] Martinez J.C., Otley C.C., Stasko T., Euvard S., Brown C., Schanbacher C.F., Weaver A.L. (2003). Defining the clinical course of metastatic skin cancer in organ transplant recipients: A multicenter collaborative study. Arch. Dermatol..

[37] Zwald F.O., Brown M. (2011). Skin cancer in solid organ transplant recipients: Advances in therapy and management. Part II: Management of skin cancer in solid organ transplant recipients. J. Am. Acad. Dermatol..

[38] Leibovitch I., Huilgol S.C., Selva D., Hill D., Richards S., Paver R. (2005). Cutaneous squamous cell carcinoma treated with Mohs micrographic surgery in Australia I. Experience over 10 years. J. Am. Acad. Dermatol..

[39] Jung J.W., Overgaard N.H., Burke M.T., Isbel N., Frazer I.H., Simpson F., Wells J.W. (2015). Does the nature of residual immune function explain the differential risk of non-melanoma skin cancer development in immunosuppressed organ transplant recipients?. Int. J. Cancer.

[40] Delgoffe G.M., Kole T.P., Zheng Y., Zarek P.E., Matthews K.L., Xiao B., Worley P.F., Kozma S.C., Powell J.D. (2009). mTOR differentially regulates effector and regulatory T cell lineage commitment. Immunity.

[41] Araki K., Turner A.P., Shaffer V.O., Gangappa S., Keller S.A., Bachmann M.F., Larsen C.P., Ahmed R. (2009). mTOR regulates memory CD8 T cell differentiation. Nature.

[42] Euvrard S., Morelon E., Rostaing L., Goffin E., Brocard A., Tromme I., Broeders N., del Marmol V., Chatelet V., Dompmartin A. (2012). Sirolimus and secondary skin-cancer prevention in kidney transplantation. New Engl. J. Med..

[43] O’Neill J.O., Edwards L.B., Taylor D.O. (2006). Mycophenolate mofetil and risk of developing malignancy after orthotopic heart transplantation: analysis of the transplant registry of the International Society for Heart and Lung Transplantation. J. Heart Lung Transplant..

